# A novel in vitro assay to predict neonatal Fc receptor-mediated human IgG half-life

**DOI:** 10.1080/19420862.2015.1054585

**Published:** 2015-05-27

**Authors:** Colby A Souders, Stuart C Nelson, Yang Wang, Andrew R Crowley, Mark S Klempner, William Thomas

**Affiliations:** MassBiologics of the University of Massachusetts Medical School; Boston, MA USA; § Current affiliation: Molecular and Cellular Biology Program; Dartmouth College, Hanover, NH, USA

**Keywords:** biolayer interferometry, correlation, glycosylation, half-life, in vitro, IgG monoclonal antibody, mutations, neonatal Fc receptor (FcRn), pharmacokinetics (PK), transgenic mice

## Abstract

Immunoglobulin G (IgG) has an unusually long serum half-life in comparison to proteins of a similar size. It is well-known that this phenomenon is due to IgG's ability to bind the neonatal Fc receptor (FcRn) in a pH-dependent manner. FcRn binding properties can vary among IgGs, resulting in altered in vivo half-lives, and therefore it would be beneficial to accurately predict the FcRn binding properties of therapeutic IgG monoclonal antibodies (mAbs). Here we describe the development of an in vitro model capable of predicting the in vivo half-life of human IgG. Using a high-throughput biolayer interferometry (BLI) platform, the human FcRn association rate at acidic pH and subsequent dissociation rate at physiological pH was determined for 5 human IgG1 mAbs. Comparing the combined FcRn association and dissociation rates to the Phase 1 clinical study half-lives of the mAbs resulted in a strong correlation. The correlation was also verified in vivo using mice transgenic for human FcRn. The model was used to characterize various factors that may influence FcRn-mAb binding, including mAb variable region sequence differences and constant region glycosylation patterns. Results indicated that the complementarity-determining regions of the heavy chain significantly influence the mAb's FcRn binding properties, while the absence of glycosylation does not alter mAb-FcRn binding. Development of this high-throughput FcRn binding model could potentially predict the half-life of therapeutic IgGs and aid in selection of lead candidates while also serving as a screening tool for the development of mAbs with desired pharmacokinetic properties.

## Abbreviations

mAb: monoclonal antibody
PK: pharmacokinetic
IgG: immunoglobulin G
FcRn: neonatal Fc receptor
β_2_m: β2-microglobulin
Fc: antibody constant region
Fab: antibody antigen binding fragment
hFcRn: human FcRn
BLI: biolayer interferometry
CDRs: complementary determining regions
VH: antibody heavy chain variable region
Vk: antibody light chain variable region
k_on_: association rate
k_off_: dissociation rate
K_D_: equilibrium affinity
CHO: Chinese Hamster Ovarian cells
ELISA: enzyme-linked immunosorbent assay
PNPP: para-nitrophenylphosphate

## Introduction

Since the first approval of products in the late 1980s and early 1990s, monoclonal antibodies (mAbs) have developed into the highest earning category of biological therapeutics.^1^ While inflammatory disorders and cancer remain the main targets, mAb therapies are being considered for a variety of new indications and an increasing number of mAbs are receiving orphan drug status.^2-4^ To meet the demand for mAbs to new targets and maintain competitiveness within the sector, novel discovery platforms and antibody engineering are required to develop the next generation of mAbs.^3,5-7^ The pharmacokinetic (PK) properties of a mAb is widely regarded as a critical parameter to optimize for the successful production of more effective, safer and cheaper mAb therapeutics.^5,8-12^

Fortunately, the last decade of research has enabled better understanding of biochemical and biophysical properties that can significantly influence mAb PK.^13^ Aside from target-mediated clearance, which contributes to non-linear PK profiles and has limited opportunity for modulation,^14-16^ the properties affecting linear and non-linear kinetics may include charge variation, nonspecific binding, size, delivery route, immunogenicity, post-translational modifications, hydrophobicity and proteolysis.^6,17-25^ However, the ability for mAbs of the immunoglobulin G (IgG) isotype to bind the neonatal Fc receptor (FcRn) in a pH-dependent manner is the critical property associated with an IgG's linear kinetic profile and extends the half-life of a typical IgG1 to, on average, 21 d.^26-28^ Functional FcRn, a heterodimeric complex of α-chain and β_2_-microglobulin (β_2_m),^29^ was first discovered when the ability of IgG to transfer across the placental barrier from mother to fetus was observed.^30-32^ Later, FcRn expression in the vascular endothelium and bone marrow-derived cells was attributed to the mechanism for IgG recycling and protection from lysosomal degradation.^33-36^ The details of this mechanism have been widely investigated.^26,37-39^ It is now well known that circulating IgGs are internalized by fluid phase pinocytosis and bind membrane-bound FcRn inside the acidified sorting endosomal compartments. Transport back to the cell surface and dissociation from FcRn at physiological pH rescues the IgG from catabolism and distributes it back to the circulation.

Elucidation of the crystal structure of FcRn^40-42^ provided a means to investigate the IgG pH-dependent binding properties in detail and, as a result, a number of studies have defined the critical amino acid contacts.^43-47^ More recently, mutations in these residues have exemplified the complex binding relationship of FcRn and IgG that modulates in vivo half-life, while highlighting the importance of both association at acidic pH (≈6.0) and dissociation at physiological pH (≈7.4).^11,18,23,37,38,48-55^ Independent of the direct mAb-FcRn contact within the IgG constant region (Fc), additional studies have determined the antigen binding fragment (Fab) can have a significant effect on the mAb-FcRn interaction and in vivo half-life.^25,56,57^ This phenomenon explains why circulating IgGs with identical Fc regions can vary 2-3 fold in serum half-life even when the same antigen is targeted.^58-60^

Despite the well-characterized mAb-FcRn interaction and its effect on in vivo half-life, several hurdles that limit the usefulness of this information in experimental testing still remain. For example, several studies have attempted to measure the mAb-FcRn interaction in vitro and draw a correlation to in vivo half-life, but the results have been largely mixed.^24,25,37,50,52,55,56,61-64^ This creates a dependency on the available transgenic FcRn mice for an accurate model of preclinical PK assessment,^22,64,65^ which is lower throughput and more costly when compared to in vitro assays. Thus, a better assay is needed to support the fast-paced mAb discovery platforms currently in use. Here we report a novel high-throughput in vitro model capable of predicting the human half-life of naturally occurring, fully human IgG mAbs. This model performs similar to in vivo models with transgenic FcRn mice, and was used to predict features that may influence mAb half-life, including complementarity-determining regions (CDRs) and Fc region glycosylation.

## Results

### Production of human FcRn and pH-dependent binding to human IgG1

**Figure 1.**
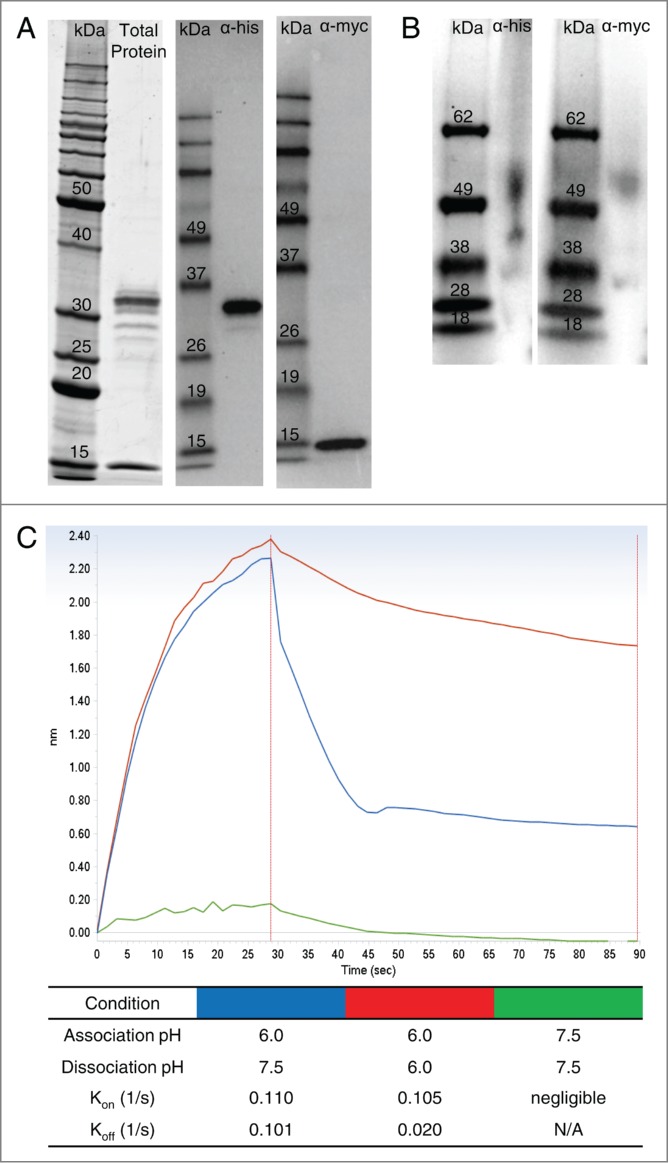
Production and characterization of functional human FcRn heterodimer. SDS-PAGE and western blot under reducing and denaturing conditions (**A**) of purified recombinant hFcRn revealed pure material at the expected molecular weight (α-chain with his-tag = 32 kDa; β_2_m with myc-tag = 16 kDa). Western blot under native conditions (**B**) indicated the purified protein is in the expected α-chain/β_2_m heterodimer form at approximately 52 kDa. Interaction of the recombinant FcRn and a human IgG1 mAb (CDA1) is pH dependent (**C**). At acidic pH (6.0), CDA1 rapidly associated (k_on_) with FcRn, but was undetectable at physiological pH (7.5). Conversely, dissociation (k_dis_) of the mAb-FcRn complex was rapid at physiological pH, but slow at acidic pH.

Human FcRn (hFcRn) was produced in Chinese hamster ovary (CHO) cells (α-chain with C-terminal His-tag and β_2_m with C-terminal myc-tag) and purified with IgG Sepharose to isolate hFcRn in its functional heterodimer form. Analysis by gel electrophoresis and western blot under reducing and denaturing conditions showed the recombinant FcRn was >90% pure with both α-chain and β_2_m expressed in equal proportions at the expected molecular weight (**Fig. 1A**). In addition, western blots under native conditions showed that >85% of the purified recombinant protein was associated as an α-chain/β_2_m heterodimer at the expected molecular weight (**Fig. 1B**).

To verify the mAb-FcRn binding properties in a biolayer interferometry (BLI)-based assay format, an hFcRn-loaded surface was incubated with a human IgG1 antibody as the analyte at acidic pH (6.0) or physiological/slightly basic pH (7.5). Binding and dissociation rates were analyzed as described in Materials and Methods. Incubation at acidic pH resulted in rapid association (k_on_ = 0.110 1/s), while physiological pH prevented any measurable binding (**Fig. 1C**). Following association at acidic pH, the mAb-FcRn complex dissociated rapidly at pH 7.5 (k_off_ = 0.101 1/s), while incubation at pH 6.0 resulted in a 5-fold slower off-rate (**Fig. 1C**).

### Correlation of human half-life to FcRn binding rate

**Figure 2.**
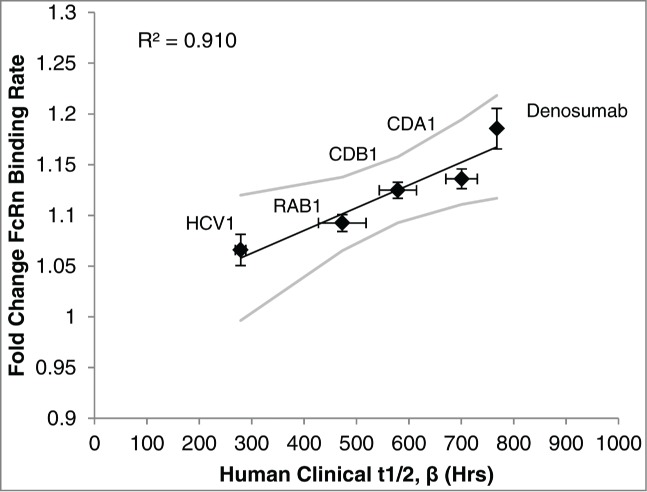
FcRn binding rate determined in vitro was compared to the human half-life previously determined in Phase 1 clinical studies. Using the BLI platform described in Materials and Methods, the fold change FcRn binding rate of 5 human IgG1 mAbs was independently determined and plotted against its known human half-life to establish a linear correlation. Values are the mean ± SEM. Line of best fit is shown in black with 95% confidence interval in gray. For in vitro experiments the average of at least 3 independent experiments is shown (N = 5 within each independent experiment). See Materials and Methods for details of clinical data.

Both association and dissociation rates are important for FcRn-mediated recycling of IgG.^25^ As such, we measured the association rate of the IgG-FcRn complex at acidic pH (6.0), followed by the dissociation rate at physiological pH (7.5) of 5 human IgG1s with a wide dynamic range of half-lives (∼12 to 32 days). The mAb association and dissociation rates were independently converted to fold change values relative to one another, and then the association and dissociation fold change values of a mAb were averaged to obtain the “fold change FcRn binding rate” for the given mAb. For example, CDA1 had an average association rate of 0.2087 ± 0.0036 1/s (± standard deviation), which was 1.0905 ± 0.0289 fold higher than the association rate of the lowest mAb assessed (RAB1), and an average dissociation rate of 0.0906 ± 0.0034 1/s, which was 1.1814 ± 0.0282 fold higher than the dissociation rate of the lowest mAb assessed (CDB1). Therefore, the average fold change FcRn binding rate for CDA1 was 1.1359 ± 0.0193. As seen in **Figure 2**, plotting the experimentally derived fold change FcRn binding rate against the β phase half-life determined previously in Phase 1 clinical studies yielded a strong linear correlation (R^2^ = 0.910).

Furthermore, the linear-curve fit model of the FcRn binding rate vs. human clinical half-life was exploited to back-calculate the predicted half-life of the mAbs in the in vitro model. The predicted half-life was within ∼10% of the clinical half-life for each mAb, and was within the error of the clinical study data (**Table 1**).

**Table 1.** Antibody half-lives predicted in vitro and clinically determined mAb half-lives

mAb	Predicted t_1/2, β_ (±SD )	Clinical t_1/2, β_ (±SD )
Denosumab	849 (±126 )^bcr^	768
CDA1	627 (±86 )^br^	701 (±181 )^abr^
CDB1	577 (±70 )^cdr^	579 (±194 )^ac^
RAB1	433 (±74 )^abd^	473 (±136 )^ac^
HCV1	314 (±138 )^abd^	279 (±55 )^abr^

Superscripts represent significant differences among mAbs within each half-life measurement condition (Predicted or Clinical): ^a^p < 0.05 vs. CDA1, ^b^p < 0.05 vs. CDB1, ^c^p < 0.05 vs. HCV1, ^d^p < 0.05 vs Denosumab, ^r^p < 0.05 vs. RAB1.

### Predicting half-life of human mAbs

**Figure 3.**
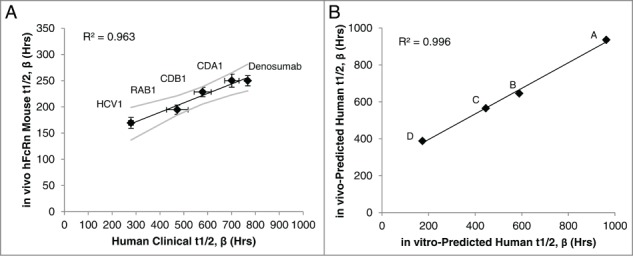
IgG1 half-life in hFcRn transgenic mice to predict human half-life. The β phase half-life of the 5 standard mAbs in **Figure 2** was assessed in hFcRn mice, which correlated to the actual human half-life determined in clinical studies (**A**). Linear regression analysis of the mAbs with known human half-life provided a line of best fit equation that was used to predict the human half-life of the pre-clinical experimental mAbs (denoted mAbs A-D) by applying the hFcRn mouse half-lives to the equation. The predicted human half-life using the hFcRn mouse in vivo data closely associated with the in vitro-predicted human half-life from the BLI-based assay (**B**). Values are the mean ± SEM. Line of best fit is shown in black with 95% confidence interval in gray. For mouse studies, 2–3 independent experiments were performed with N = 6 mice per experiment. In vitro results represent at least 2 independent experiments with N = 5 per experiment.

Given the predictive power of our model based on the 5 antibodies in **Figure 2**, we assessed the ability of the predictive model to estimate half-life of antibodies in pre-clinical studies. The four lead mAbs of an ongoing project (mAbs A, B, C and D) were assessed using the in vitro model, as well as in vivo using hFcRn transgenic mice. The five standard mAbs (Denosumab, CDA1, CDB1, RAB1 and HCV1) were also assessed in vivo to verify the accuracy of the hFcRn mouse model and create a standard curve to calculate the in vivo predicted human half-life. In agreement with previous literature reports,^22,25^ a strong positive linear correlation (R^2^=0.963) was detected between the observed half-life in hFcRn mice and the reported human half-life in clinical studies (**Fig. 3A**).

Based on the in vivo linear correlation between transgenic murine and human half-life, the line of best fit equation of the standard mAbs (**Fig. 3A**) and experimentally derived hFcRn mouse half-life was used to calculate the in vivo model prediction of human half-life for the 4 experimental mAbs. Similarly, the in vitro assay line of best fit equation from **Figure 2** and experimentally derived fold change FcRn binding rate of the experimental mAbs was used to calculate the in vitro model predicted human half-life. The in vivo and in vitro model predictions closely associated with one another (R^2^ = 0.996; **Fig. 3B**), indicating both models predicted relative differences among the experimental mAbs similarly. Absolute values of the in vivo and in vitro predicted half-lives for the experimental mAbs plotted in **Figure 3B** are shown in **Table 2**, and also agree between the 2 models.

**Table 2.** Predicted human half-life of preclinical experimental mAbs using in vivo and in vitro models

		Predicted Human t_1/2, β_ Using:	
mAb	in vivo model (Hrs)	in vitro model (Hrs)	
A	936	963
B	646	590
C	565	446
D	388	174

### Predicting half-life of modified antibodies

#### Primary amino acid sequence

To assess the ability of our in vitro predictive model to estimate the half-life of a given mAb following changes to the primary amino acid sequence, we decided to transplant the CDRs of one mAb (donor mAb) into the framework of another mAb (acceptor mAb) for the heavy (VH) or light chain (Vk). Several factors were considered for this experiment: 1) The donor and acceptor mAb had identical Fc region sequences; 2) The VH or Vk regions were of the same gene family, but contained several framework amino acid differences (13 for VH – 7 of which are similar amino acids; and 6 for Vk – 3 of which are similar amino acids); 3) The wild type donor and acceptor mAbs have significantly different half-lives in both Phase 1 clinical studies and the predictive model; and 4) The overall charge (specifically the pI) of the mAb was unchanged after exchanging the CDRs. Following these conditions, we could accurately predict the Fab region that influences the half-life of a mAb: the VH CDRs, VH framework mutations, Vk CDRs or Vk framework mutations.

**Figure 4.**
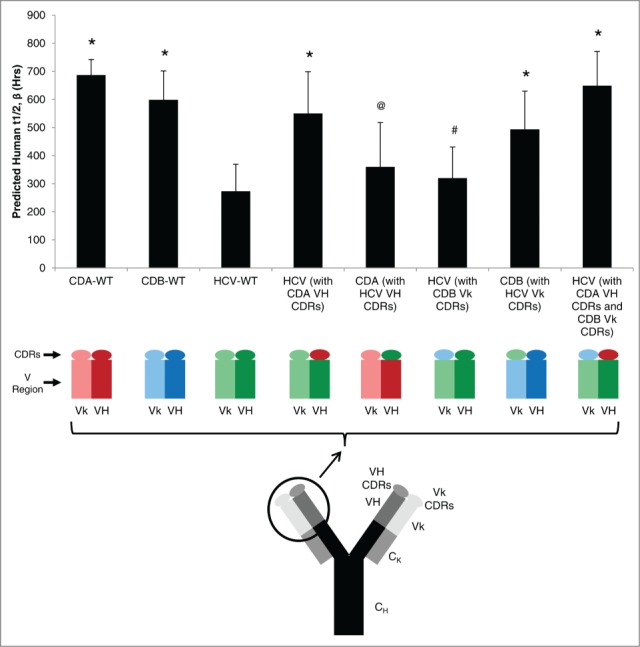
Exchanging CDRs between mAbs to modulate FcRn-binding properties. Three wild type (WT) antibodies have distinct half-lives determined from clinical studies and were predicted using the in vitro BLI-based platform. Substitution of the 3 heavy chain (VH) complimentary determining regions (CDRs) significantly changed the predicted half-life of the mutant mAb to resemble the predicted half-life of the CDRs' parent mAb. Conversely, substitution of light chain (Vk) CDRs did not affect the predicted half-life. Substitution of both VH and Vk CDRs produced a mAb with a predicted half-life similar to the original half-life of the CDR parent mAb. Mean ± SD is reported of at least 2 independent experiments, with N = 5 for each experiment. Significant differences among mAb constructs are denoted by: *p < 0.05 vs. HCV-WT; ^@^p < 0.05 vs. CDA-WT; ^#^p < 0.05 vs. CDB-WT.

Analysis using our in vitro predictive model indicated that inserting the VH CDRs of a mAb with a naturally longer half-life (CDA1) into the VH framework of a mAb with a significantly shorter half-life (HCV1) created a mutant mAb with a half-life predicted to be similar to the donor and significantly longer than the acceptor mAb (**Fig. 4**). The converse was also true, where insertion of VH CDRs of HCV1 into the framework of CDA1 created a mutant mAb with a predicted half-life similar to HCV1 and significantly shorter than CDA1 (**Fig. 4**).

However, the influence of CDR sequence was not observed when the same approach was taken with Vk CDRs. In this case, inserting Vk CDRs of a mAb with a naturally longer half-life (CDB1) into the Vk framework of a mAb with a significantly lower half-life (HCV1) created a mutant mAb with a half-life similar to the acceptor mAb and significantly shorter than the donor mAb (**Fig. 4**). Again, the converse was true whereby the predicted half-life of the mutant mAb was significantly longer than the donor mAb following Vk substitution with CDRs from a mAb with a shorter half-life. Finally, creating a double mutant by inserting both VH and Vk CDRs from mAbs with longer half-lives into a mAb with a shorter half-life indicated the half-life of the double mutant adopted that of the donor mAbs (**Fig. 4**). Taken together, these experiments indicate that the VH CDRs represent the Fab region that significantly affects the half-life of a given mAb.

#### Post-translational modifications

**Figure 5.**
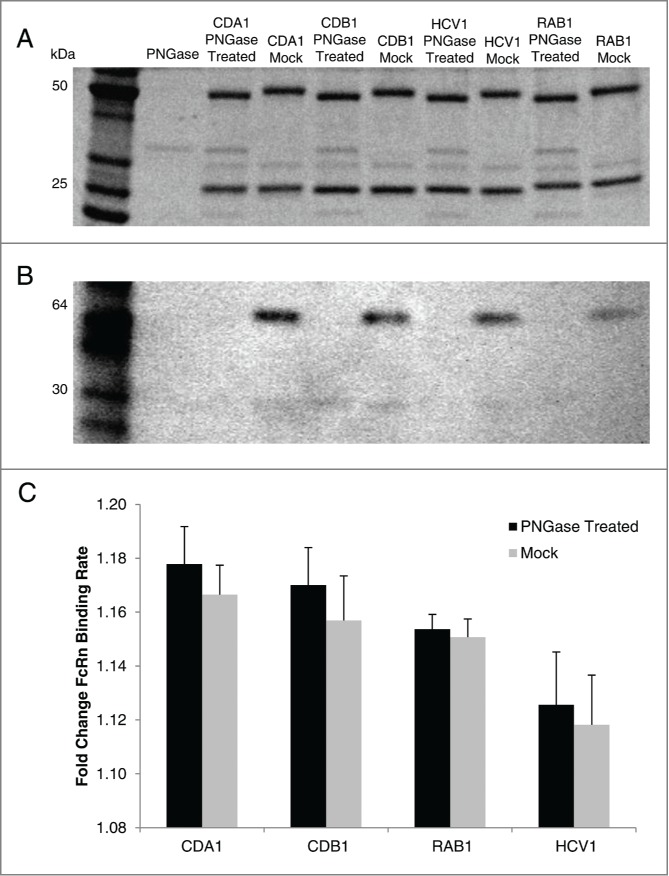
FcRn binding properties of glycosylated or aglycosylated mAbs. PNGase treatment of 4 human IgG1 mAbs expressed in CHO cells produced aglycosylated versions that, when compared to mock treated mAb, was confirmed by size shift on SDS-PAGE (**A**) and lack of staining by Concanavalin A on western blot (**B**). Using the in vitro BLI-based assay to assess mAb-FcRn interactions, the glycosylated (mock) and aglycosylated (PNGase treated) version had similar binding rates in each case (**C**). Mean ±SD is reported for 3 independent experiments with N = 5 for each experiment.

Modification of glycan structure is a common posttranslational event in vitro and in vivo. It has been reported that the absence of an Fc glycan in the germline C_H_2 domain does not alter the half-life of antibodies,^66-69^ and thus we were interested in confirming this conclusion with our predictive model. Following PNGase treatment, 4 antibodies were confirmed to be aglycosylated by size shift and western blot analysis (**Fig. 5A, B**). Comparison of hFcRn binding rates using the in vitro predictive model indicated no significant difference between glycosylated and aglycosylated mAb species (**Fig. 5C**), confirming that this model is capable of portraying effects (or lack thereof) from post-translational modification.

## Discussion

PK properties are often critical to the success of a therapeutic, and recent reports have suggested ∼15% of Phase 1 clinical studies fail due to inadequate PK or pharmacodynamics properties.^70^ This is especially true for antibodies, where typically longer half-lives are desired for therapeutics to decrease dosing frequency and increase efficacy. Conversely, a short half-life may be desired for in vivo diagnostic mAbs where toxicity and off-target effects should be reduced. Here, we developed a novel in vitro, high-throughput screening method for predicting antibody half-life using a BLI platform.

Initially, high quality, fully functional human α-FcRn/β_2_m (hFcRn) was purified and characterized by its ability to bind human IgG1 in a pH-dependent manner. As expected, and consistent with literature reports,^28,55,57^ a human monoclonal IgG1 associated quickly with hFcRn at a slightly acidic pH, while negligible binding was detected at physiological pH. Furthermore, dissociation of the mAb-hFcRn complex was slow at acidic pH and rapid at physiological pH. Similar to previous studies that conveyed the importance of high-quality hFcRn and assay format,^57^ our results indicated the hFcRn we used is of high quality, and functions similar to its in vivo mechanism when positioned correctly as the ligand in a BLI platform. Neuber et al.^57^ previously verified the accuracy of the BLI platform in comparison to surface plasmon resonance-based assays and highlighted the importance of further developing predictive models.

To establish a predictive model that considers both association and dissociation of the mAb-FcRn complex, a BLI kinetic model was used to calculate the association rate (k_on_) at acidic pH followed by the dissociation rate (k_off_) at physiological pH for 5 different antibodies with known half-lives ranging from ∼12 to ∼32 days; a generally acceptable dynamic range for therapeutic mAbs. A strong correlation between the FcRn binding rate and the human half-life determined from Phase 1 clinical studies was observed. Specifically, mAbs with a fast on or off rate had a longer half-life. Initial analysis indicated that both association and dissociation should be considered, as neither condition alone could positively correlate to the known human half-life (data not shown). These results may explain why some studies struggled to find a correlation between pH 6.0 equilibrium affinity and in vivo half-life.^24,37,50,52,56,61-64^ Some predictive models have suggested mAb-FcRn equilibrium affinity (K_D_) at acidic pH is insufficient to accurately account for changes in half-life, and instead the rate of association should be considered.^49^ Furthermore, other literature reports stress the importance of assessing dissociation characteristics at physiological pH to more accurately predict half-life.^25,37,55,63^ It is likely that both the rates of association in the endosome and extracellular dissociation of the mAb-FcRn complex are critical for recycling. Our results support the use of on/off rates rather than equilibrium binding while considering the association and dissociation at relevant pH to accurately predict in vivo half-life.

To verify our model and extend its usefulness, we determined the predicted human half-life using the in vitro model for 4 lead human IgG1 mAbs still in preclinical studies, and compared the prediction to their in vivo half-life using hFcRn transgenic mice. This mouse model is widely accepted as an accurate surrogate for determining relative human half-lives of IgGs,^22,25^ and our model confirmed the correlation between human and transgenic mouse half-life for the 5 mAbs tested with human clinical study data. Furthermore, comparing the estimated human half-life extrapolated from the in vivo study to the in vitro predicted half-life yielded a very strong positive correlation for the mAbs within a half-life range of ∼12–40 d Not only did the 4 antibodies have the same rank order in both assays, but the absolute predicted half-life varied by only about 3–20% between the 2 predictions for 3 of the 4 mAbs. Taken together, these results suggest the in vitro assay described here is capable of predicting the human half-life of mAbs similar to hFcRn transgenic mice.

A reliable, high-throughput assay that can predict the human half-life of a mAb after it has under gone modifications would be beneficial. To assess this possibility, and to address the question of what Fab region influences mAb half-life,^25,56,57^ we decided to substitute CDR regions of one mAb with that of another. The in vitro assay was able to predict significantly different half-lives in select mutants and supported the hypothesis that heavy chain CDRs represent the region that influences mAb half-life as opposed to framework mutations or light chain CDRs. This expands similar investigations by Neuber et al.^57^ who assessed the K_D_ at acidic and physiological pH of a panel of mAbs to determine that the different germline immunoglobulin gene families did not influence FcRn binding. They further concluded that CDR1 and CDR2 regions also had no effect, and proposed that VH/VL framework mutations or the CDR3 sequence was responsible for modulation of the FcRn interaction. When combined with our results, it can be inferred that the CDR3 sequence is the cause of FcRn binding variation among different mAbs with the same constant region; however, additional experimentation followed by in vivo studies are needed for confirmation. Also, given the recent finding that local charge distribution can affect mAb PK,^71^ it would be interesting to expand upon the case study presented here with a panel of mAbs containing various charge distributions in the CDR3 region.

In addition to primary amino acid sequence changes, post-translational modifications were also of interest. Early reports were conflicted regarding effects of glycosylation on antibody half-life,^18,72-75^ but our results support the most recent reports suggesting glycosylation does not affect FcRn-mediated PK.^66-69^ In our study, this was confirmed by removing all N-glycosylation (only the natural germline Fc glycosylation site exists in the antibodies tested) and the in vitro FcRn binding measurement was used to compare the aglycosylated mAb to the naturally occurring glycosylated form produced in CHO cells. This finding further suggests the in vitro model described here is capable of recapitulating data produced from more complicated, expensive and time-consuming in vivo studies that were previously required for reliable PK data. However, additional posttranslational modifications, including various glycan species, should be tested to confirm our model.

One limitation of the present study is the restriction to antibodies that bind soluble targets and are not affected by target-mediated clearance, which is expected to be a source of clearance for mAbs targeting the cell surface or other endogenous antigens.^16,22^ Since the assay only measures mAb-FcRn interactions, factors influencing mAb clearance independent of FcRn cannot be predicted and animal models typically can address some of these factors. The influence of many biophysical properties, such as pI, are well established,^17^ and standard features of the mAb can be assessed in conjunction with FcRn binding properties to gain a complete understanding of the mAb's PK properties. Indeed, others have suggested a combination of biophysical properties is responsible for a given mAb's PK profile.^19,57^ We suggest the in vitro model described here can provide a critical piece of the puzzle, given FcRn interactions are a major parameter.

The quality of antibody preparations must be considered in addition to FcRn. For example, antibodies with a propensity to aggregate or degrade must be stored appropriately because destabilization of the mAb will significantly affect its FcRn binding properties. Although the mAbs are in defined buffers when FcRn binding is measured, optimal purification and formulation buffers should be used prior to analysis. It may be possible to use the FcRn binding assay to screen purification and formulation conditions given its sensitivity, and future studies will address this property.

Additional analysis should also refine the ability of the assay to predict the half-life of mAbs with Fc mutations, engineered Fc regions or Fc fusion proteins. For example, some mutations that enhance in vivo half-life have delayed release and higher affinity at physiological pH (data not shown),^12^ which significantly affects the overall FcRn binding rate measured in this study. Regardless, select mutations have been shown to significantly enhance the half-life in humans.^76^ Conversely, some mutations that reduce half-life, including H435A, can significantly alter the FcRn association rate,^77^ making an overall FcRn binding rate measurement difficult. Modulating the assay parameters or method of calculating FcRn binding rate may allow these mutants to be accurately assessed using this model.

In summary, we developed a high throughput in vitro assay to predict the FcRn-mediated aspect of half-life for human IgG1 antibodies in humans. The assay is comparable to hFcRn transgenic mice in predictive power, yet it takes only 1 to 2 hours for full analysis of multiple mAbs, costs a fraction of the operating cost and requires limited mAb quantities (<0.5 mg for multiple experiments). Using the model, we found that the VH CDRs represented the Fab region that influences mAb half-life, and also confirmed recent studies claiming aglycosylated mAbs have similar half-lives compared to their glycosylated form. Further development of the model should allow additional properties of mAbs to be assessed with precision, including stability and the effects of Fc point mutants, among others.

## Materials and Methods

### Human FcRn production

Genes encoding the soluble ectodomain of human FcRn α-chain (amino acids 34–290; GenBank Accession #AAH0734.1) and full-length β_2_m (GenBank Accession #AAH64910.1) without the signal peptide were codon optimized for synthesis (Genewiz). A C-terminal His_6_ tag or a C-terminal Myc tag was cloned in frame with the α-chain or β_2_m proteins, respectively. Subsequently, a single vector was produced for the simultaneous expression of soluble human αFcRn and β_2_m in CHO cells, suspension cells adapted from CHOK1 (ATCC, CCL^−^61).

The full length plasmid containing α and β subunits was stably transfected in CHO cells via electroporation using the Gene Pulser Xcell system (BioRad). Soluble FcRn expression was assessed by anti-tag enzyme-linked immunosorbent assays (ELISA) and the 8 highest expressing CHO cell clones during early growth were pooled and scaled. The FcRn-containing media was collected, centrifuged and filtered using 0.2 µm filters. The heterodimeric, functional FcRn protein was purified from the media using IgG Sepharose 6 Fast Flow (GE Healthcare, 52–2083–00) on an Econo Column (BioRad, 737–2522). Based on the pH-dependent affinity of FcRn to IgG, columns were washed with PBS pH 6.0 and FcRn eluted with PBS pH 8.0. Fractions were concentrated with centrifugal concentrators (Pierce, Thermo Scientific, 89885A) and purified FcRn was stored at −80°C as single-use aliquots.

### Characterization of purified hFcRn

FcRn protein purity was analyzed by SDS-Tris-glycine polyacrylamide gel electrophoresis (Life Technologies) under reducing conditions followed by SYPRO ruby staining (Life Technologies) using the manufacturers' recommended protocol. FcRn protein quality was also analyzed by Western blot under native conditions using Tris-Bis NativePage (Life Technologies) or reduced and denatured conditions using SDS-Tris-glycine polyacrylamide gels and transferred onto PVDF membranes (EMD Millipore). Using standard Western blot procedures, FcRn was probed with either a mouse anti-myc or a mouse anti-his antibody, detected by a secondary anti-mouse-HRP antibody (Jackson ImmunoResearch) and developed with Amersham ECL Western Blotting Detection Reagent (GE Healthcare, RPN2106). All gels and blots were imaged on a ChemiDoc MP (BioRad) and band intensity and molecular weight was estimated with Image Lab Software (BioRad).

### Monoclonal antibodies

All mAbs used for in vitro and in vivo predictive models are fully human IgG1s that bind an exogenous or endogenous soluble target in vivo. The standard mAbs used to generate curve fit models for the in vitro and in vivo predictive model have reported human in vivo half-life data in healthy individuals. Of these 5 mAbs, 4 were produced by MassBiologics:^78-80^ CDA1 (i.e. actoxumab; Merck), CDB1 (i.e., bezlotoxumab; Merck), RAB1 and HCV1. Denosumab was produced by Amgen.^81^ All mAbs were produced under GMP conditions with a CHO cell manufacturing cell line. The four pre-clinical mAbs are also human IgG1s that bind an exogenous soluble target and were manufactured, purified and formulated by MassBiologics under GMP-like conditions using similar methods as the clinical mAbs.

### BLI-based assessment of mAb-FcRn interactions

The Octet QK (PALL/ForteBio) was used for all FcRn binding in vitro assays at 30°C in 96-well solid black plates (Greiner Bio-One, 655900). Initially, FcRn was immobilized to nickel-nitrilotriacetic acid -coated biosensors (ForteBio, 18–0029) for 180 seconds at an optimized concentration. After a baseline step, the mAb-FcRn binding rate was determined when the biosensor with immobilized FcRn was exposed to the mAb sample in PBS adjusted to pH 6.0 with HCl for 30 seconds. Following association, the mAb-FcRn complex was exposed to PBS pH 7.5 and the rate of mAb dissociation from FcRn was measured. Prior to analysis, all antibodies were dialyzed into PBS pH 6.0, diluted to 100 µg/ml in PBS pH 6.0 and used at a 200 µl volume. Each assay is performed on a specific mAb in quintuplicate. Data analysis was performed using software version 7.0 (PALL, ForteBio). In a step specific manner, the given K_on_ and K_off_ rates were determined using the first 20 and 30 seconds of the association and dissociation steps, respectively. Savitzky-Golay filtering was applied to increase signal-to-noise ratio and rates were calculated with R equilibrium steady-state fitting. Rates were reported in 1/s and the mean K_on_ and K_off_ values of each mAb within an experiment were individually normalized to fold-change values among all tested mAbs. The fold-change FcRn binding rate was determined by averaging the mean fold-change K_on_ and mean fold-change K_off_ values of a mAb. The mAb FcRn binding rates and the accompanying known human half-lives from human Phase 1 clinical study data were used to generate a linear regression best-fit correlation.

### hFcRn mice in vivo half life prediction

Human FcRn transgenic mice used in this study were hemizygous B6.Cg-Fcgrt^tm1Dcr^ Tg(FCGRT)32Dcr/DcrJ from The Jackson Laboratory (Stock #014565). These animals lack the endogenous mouse FcRn α gene and express the human FcRn α gene on one allele under control of the native FcRn promoter. The animals have the native mouse β_2_m gene. The specific strain was selected based on a previous study examining the different strains.^22^ Female mice of 3 different age groups were used in this study: 8, 14 and 20 weeks old.

For PK studies, mice received a single intraperitoneal injection containing a mixture of IgGs such that each mAb was administered at 1 mg/kg. Each mixture was composed of the 5 mAbs with known human half-life (denosumab, CDA1, CDB1, HCV1 and RAB1) and 4 or 5 different pre-clinical mAbs. All mAbs within a mixture could be individually identified by specific antigen binding. Serial submandibular bleeds were collected in Microtainer Serum Separator Tubes (BD, 365956) at 2 hours, 1 day, 7 days, 14 days, 21 days, 29 d and 41 d after injection. Animals were separated into subgroups to stagger early time point bleeds. Blood samples were centrifuged at 3500 rpm for 10 minutes and the serum fraction was collected and stored at −80°C until use.

The concentration of each human IgG in mouse serum samples at the various time points was determined by ELISA. Briefly, 96-well High-binding ELISA plates (Costar, 07–200–39) were coated with an optimized concentration of the mAb-specific antigen in PBS and blocked with 1% Bovine Albumin Fraction V (MP Biomedicals, 810034) in PBS + 0.05% Tween20 (Sigma-Aldrich, P1379). Mouse sera samples and standards (mAb mix used for injections or single mAb alone) were serially diluted in blocking buffer and incubated on antigen-coated plates for 1 hour. Bound hIgG was detected with Alkaline Phosphatase-conjugated AffiniPure Goat Anti-Human IgG (Jackson ImmunoResearch, 109–055–098) after developing with para-nitrophenylphosphate (PNPP) (Thermo, 34047). Optical density (OD) was measured at 405nm on a Precision Microplate Reader (Molecular Devices) and mean mAb concentration of test samples were determined from the standard curve using a 4-parameter nonlinear regression model in Softmax Pro software (Molecular Devices). Extrapolated concentrations were multiplied by sera dilution factors and the mean value was used to determine final IgG concentrations.

To identify mice that mounted an anti-hIgG immune response during the experiment, plates were coated with the mAb mix used for injection diluted in PBS to 1µg/mL per mAb. Plates were blocked and sera samples from day 21 to 41 time points were serially diluted and added to the plate. Bound murine IgG was detected with Alkaline Phosphatase-conjugated AffiniPure Goat Anti-Mouse IgG (Jackson ImmunoResearch, 115–055–003) after developing with PNPP (Thermo, 34047). OD was measured at 405nm on Precision Microplate Reader (Molecular Devices) and mice with ODs 5-fold background levels were considered to have a significant anti-hIgG response and were removed from data analysis.

Half-life calculations were determined using Phoenix WinNonlin 6.3 (Certara) noncompartmental analysis. Consistent time points representing the β-phase half-life (7–41 d post injection) were analyzed using the linear trapezoidal linear interpolation calculation method. This study was carried out in strict accordance with the recommendations in the Guide for the Care and Use of Laboratory Animals of the National Institutes of Health. The protocol was approved by the Institutional Animal Care and Use Committee of the University of Massachusetts Medical School (Protocol Number: A-2412).

### CDR-exchanged mAbs

Mutant mAb gene sequences were constructed using the IMGT database to identify CDR region nucleotides within the heavy and light chain variable regions. Differences between mAbs in the framework region primary amino acid sequence were identified using the alignment tool in Vector NTI (Life Technologies) with the default Similarity Table settings. Mutant sequences were constructed such that the variable region contained the framework of one mAb (denoted the “acceptor” mAb) and the CDR1, 2 and 3 of a different mAb (denoted the “donor” mAb). The modified genes were synthesized (Genewiz) and heavy and light chain genes were transiently co-transfected in 293T Human Endothelial Kidney Cells (ATCC, CRL-11268) with 25 kDa linear polyethylenimine (Polysciences). The media was collected 6 d after transfection and centrifuged to remove cell debris. The IgG-containing media was filtered using 0.2 µm filters (Thermo Scientific, Nalgene), purified by standard Protein A sepharose affinity methods and eluted with 100 mM glycine pH 2.8. Purified mAbs were dialyzed into PBS pH 6.0 and stored at 4°C until use.

### Aglycosylated mAbs

Four mAbs produced by MassBiologics (CDA1, CDB1, HCV1, and RAB1) were treated with PNGase F (New England BioLabs) to remove N-linked glycosylation. For each antibody, 100µg was incubated with 6µl of PNGase F for 3 hours at 37°C. A mock control reaction was performed with 6µl of PNGase buffer instead of enzyme. The product was analyzed by SDS-Tris-glycine PAGE (Life Technologies) under reducing/denaturing conditions and SYPRO ruby stained (Life Technologies) using the manufacturers' protocol. The absence of N-linked glycan was also analyzed by Western blot after transfer to PVDF membrane (EMD Millipore). The blot was probed with a 1µg/ml solution of HRP conjugated Concanavilin-A (Sigma-Aldrich) and developed with Amersham ECL Western Blotting Detection Reagent (GE Healthcare, RPN2106).
